# Assessment of Reporting Bias for *Clostridium difficile* Hospitalizations, United States

**DOI:** 10.3201/eid1408.080446

**Published:** 2008-08

**Authors:** Marya D. Zilberberg

**Affiliations:** * University of Massachusetts, Amherst, Massachusetts, USA; † Evi*Med* Research Group, LLC, Goshen, Massachusetts, USA

**Keywords:** *Clostridium difficile*, gastroenteritis, hospitalizations, epidemiology, letter

**To the Editor**: Burckhardt et al. (*1*) recently reported on *Clostridium difficile*–associated disease (CDAD) in Saxony, Germany. In contrast to the observation by Wilcox and Fawley in the United Kingdom (*2*), the report from Germany argued against a reporting bias for gastroenteritides as a cause of the observed increase in the incidence of CDAD diagnoses from 2002 through 2006. To explore this issue further, I examined the potential influence of such reporting bias on the observed increase in the incidence of hospitalizations of patients with CDAD in the United States from 2000 through 2005.

In the 2000–2005 data from the National Inpatient Sample data from the Agency for Healthcare Research and Quality (*3**,**4*), I identified hospitalizations for gastrointestinal infections caused by *C. difficile*, *Salmonella*, rotavirus, and other unspecified infectious agents, using the corresponding diagnosis codes from the International Classification of Diseases, 9th Revision, Clinical Modification. I obtained censal and intercensal data on the numbers of the U.S. population from 2000 through 2005 from the U.S. Census Bureau (*5*). Based on these records, I calculated hospitalization incidence for each of the infectious causes.

Annual incidence of CDAD increased from 49.2 to 101.6 per 100,000 population within the period examined. Within the same time frame, the incidence of CDAD as the principal diagnosis also more than doubled, increasing from 11.6 to 25.8 hospitalizations per 100,000. Although the incidence of hospitalizations for *Salmonella* infections per 100,000 population remained stable, rotavirus infection showed a slight increase (from 10.8 to 14.5) as did other infectious gastroenteritides (from 38.9 to 49.9/100,000) (Figure). Thus, although a slight increase in the incidence was exhibited, a reporting bias for gastroenteric infections with organisms other than *C. difficile* does not appear to account fully for the observed doubling of the overall incidence of hospitalizations with CDAD in the United States from 2000 through 2005.

**Figure:**
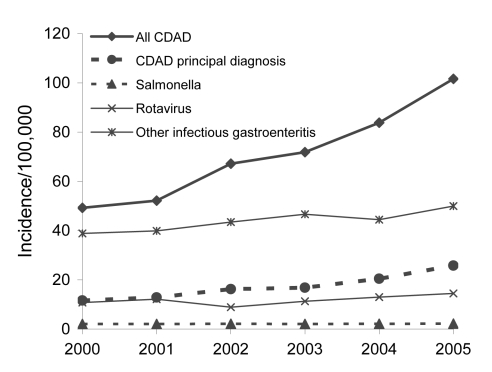
Annual incidence per 100,000 population of all hospitalizations for *Clostridium difficile*–associated disease (CDAD) compared with hospitalizations for a primary diagnosis of CDAD and with gastroenteritides caused by *Salmonella*, rotavirus, and other unspecified infectious agents, United States, 2000–2005.
